# Multi-Criteria Decision Analysis for Mechanical Recyclability Assessment of Different Types of PET Packaging Waste

**DOI:** 10.3390/polym18091063

**Published:** 2026-04-28

**Authors:** Giusy Santomasi, Francesco Todaro, Michele Notarnicola, Eggo Ulphard Thoden van Velzen

**Affiliations:** 1 Department of Civil, Environmental, Land, Building Engineering and Chemistry (DICATECh), Polytechnic University of Bari, Via E. Orabona n.4, I-70125 Bari, Italy; 2 Wageningen Food & Biobased Research, Wageningen University & Research, Bornse Weilanden 9, 6708 WG Wageningen, The Netherlands

**Keywords:** rPET, mechanical recycling, multi-criteria analysis, recyclability assessment, end of waste routes

## Abstract

The management of plastic packaging waste needs to be optimized to improve recycling rates. In this article, fourteen categories of non-bottle polyethylene terephthalate (PET) packages were mechanically recycled at laboratory bench scale; the generated data were assessed using a multi-criteria decision analysis (MCDA) approach to identify the categories most suited for the mechanical recycling process from social, technical and legislative viewpoints. Recycling yields varied between 75% and 92% across the 14 categories. The intrinsic viscosity (IV) values of the produced recycled PET (rPET) corresponded to molecular weights ranging from 28,000 to 35,000 g/mol. The MCDA recyclability assessment showed that 7 of the 14 categories (accounting for 72% of the sorted products by mass flow) are often composed of multiple, inseparable materials, resulting in the lowest-quality rPET. Furthermore, only 4 categories (approximately 28% of the categories) were found suitable for closed-loop mechanical recycling. The stakeholders involved in the PET packaging value chain could use these results to support decision-making and the development of a well-organized framework to valorize even the most complex types of plastic waste.

## 1. Introduction

The European Green Deal strategy for plastic packaging is based on simultaneously setting recycling targets, defining recyclability requirements, and demanding minimum recycled content for new products [1].

Recycling PET (polyethylene terephthalate) waste (one of the most important polymers [2]) helps to reduce fossil fuel consumption, carbon dioxide emissions and the amount of waste sent to landfills [3]. Nonetheless, mechanical recycling of PET waste constitutes the most economical, energy-efficient and ecologically efficient option [4]. For example, mechanical recycling of PET bottles results in high-quality rPET, as PET bottles are designed for recycling in Europe [5].

Although PET packaging waste flows also include PET trays, thermoformed sheets are used for other food packaging. These streams are much more heterogeneous than bottle streams in composition, including different elements, such as paper labels, lids, and multilayers such as polyethylene (PE), ethylene vinyl alcohol (EVOH) and polypropylene (PP), food residues and sorting mistakes [6,7,8].

For multilayer packaging, there is a gap in established solutions [9,10]. In the literature, there are several studies on the recycling of multilayer packaging focused on chemical processes [10,11]. Still, there are very few on mechanical recycling: Barredo et al. (2023) [12] and Ügdüler et al. (2020) [13] proved a potential route for closing the PET tray recycling loop, through alkaline hydrolysis depolymerization; Eriksen et al. (2019) [14] discovered that contamination in recycled material promotes decreased quality and increased variability of the recovered polymer; Delva et al. (2019) [15] addressed the challenges in recycling multilayer packaging materials composed of PET and PE, using compatibilizers to improve the mechanical performance of these blends.

On the other hand, mechanical recycling research has focused on different stages of the process: Küppers et al. (2019) [16] proved the influence of label removal on the sorting phase for PET bottles; Krehula et al. (2013) [17] studied the washing step for PET waste; Seier et al. (2022) [18] investigated the temperature effects on the multilayer trays during mechanical processes, also exploring the polymer composition of PET trays. However, there is a lack of research in the scientific literature on how to overcome the challenges associated with recycling PET trays. The main challenge lies in recycling heterogeneous packaging, which varies in both type and material composition, adding complexity to the process. In addition, the literature shows the complexity of plastic waste recycling, which involves an integrated process encompassing multiple aspects: technological, environmental, and socio-economic. In these cases, multi-criteria decision analysis (MCDA) provides the necessary decision-support framework to compare and select solution possibilities [19,20]. Several MCDA-based frameworks have been developed in strategic decision-making in the solid waste management area, for example: (i) to evaluate different disposal alternatives for plastic waste [21]; (ii) for strategic planning, which includes allocation of waste among treatment facilities, but also for treatment technology selection [22] and recycling technology choice [23].

This study aims to evaluate the mechanical recycling of 14 categories of PET trays (mono- and multi-material), which are used as separate feedstocks for a small-scale recycling process. Given the heterogeneity of the data, multi-criteria decision analysis (MCDA) was applied to classify the 14 different types of PET trays into three levels of compatibility with closed-loop mechanical recycling (i.e., production of transparent, food-grade rPET). For each compatibility level, the corresponding recovery pathway was identified.

The results allowed us to provide information about PET trays correlating material composition with the technical efficiency of the mechanical process, overcoming the impossibility of finding a direct correlation between them and achieving consistent outcomes useful for the different stakeholders. In the end, a hypothetical material flow was implemented to link the category to its corresponding end-of-life path, based on the technologies available in real plants.

This study could help, in particular, the research concerning: the field of eco-design, i.e., for the development of guidelines for new products complying with mechanical recycling; recycling technology studies, i.e., for the implementation of determined treatments into the process, and also for further MCDA applications (i.e., this study can be extended by adding criteria and new analyses and/or in further studies about different waste).

## 2. Materials and Methods

### 2.1. Framework of the Research

**Figure 1:**
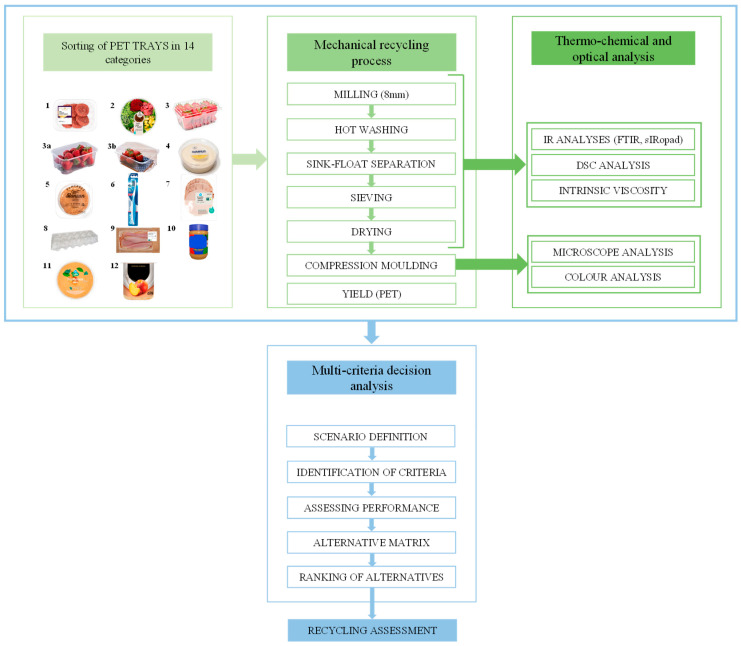
Overview of the following approach. PET tray categories: 1 = transparent meat, cheese, fish, pasta, vegetarian trays (multilayer); 2 = bowls and fresh salad trays; 3 = clamshells and top-sealed trays for fresh fruits, vegetables, and nuts; 3a = clamshells and top-sealed trays for fruit with PE bubble wrap inlay with hotmelt; 3b = clamshells and top-sealed trays with moisture absorber inlay with hotmelt; 4 = smearable salad trays; 5 = clear trays and clamshells for cookies and bakery products (mono PET); 6 = non-food blisters; 7 = thermoformed trays for cured meat products and sliced cheese; 8 = container for eggs; 9 = flat trays to support sliced meat and cheese products; 10 = jars (e.g., peanut butter, creams); 11 = loose lids and caps; 12 = pots for yogurt.

The overall research approach is illustrated in Figure 1. The PET tray waste was divided into 14 categories—based on composition and previous use (e.g., type of packed food)—and recycled separately. The recycling efficiencies were determined, and the resulting products were analyzed using a range of thermal, optical, and spectrophotometric techniques. Finally, multi-criteria decision analysis (MCDA) was employed to assess and rank the recyclability of the different categories based on the analysis results, considering social, technical, and legislative perspectives.

### 2.2. Materials

A sample of 300 kg PET tray waste [24] was collected at the sorting facility Attero (Vamweg 7, 9418 TM, Wijster, The Netherlands). This sample was manually sorted into 14 different PET tray categories (12 main categories and 2 subcategories) based on previous use (e.g., food-grade package categories), following the same procedure as in Santomasi et al. (2024) [7].

### 2.3. Methods

#### 2.3.1. Mechanical Recycling

Samples of the 14 categories were milled using a Wanner C 17.26sv knife mill (Wanner Technik GmbH, Alte Heerstrasse 5B, Wertheim-Reicholzheim, Germany) equipped with an 8 mm sieve plate. The main parameters for washing have been established based on a combination of real plant processes, literature analysis [17] and the protocol of Wageningen Research [25] for rigid PET packaging. Parameters include stirring speed, waste/water ratio, amount of detergent, and temperature.

Roughly 50 g of milled PET trays was weighed. The perfect flake: water ratio for the set-up (2 L beaker glass with mechanical top stirrer) was 1:8; thus, 400 mL of 0.25 M an aqueous solution of sodium hydroxide (NaOH; M = 40.0 g mol^−1^, ρ = 2.13 g cm^−3^, Sigma-Aldrich Chemicals (Stationsplein 4, Zwijndrecht, The Netherlands)) was used. The PET tray waste samples were washed at 85 °C for 15 min, with constant stirring at 800–1000 rpm. After washing, the solution was filtered, and PET flakes were rinsed in demineralized water to remove traces of NaOH and dirt.

Thereafter, by sink–float separation [26] the floating and the sinking fractions were collected, rinsed and dried (12 h at 105 °C in the oven) separately with plentiful demineralized water over three sieves (6 mm square mesh, 3 mm square mesh, and 0.5 round mesh) to separate the targeted material from two types of sludge.

#### 2.3.2. rPET Characterization

The washed flakes were analyzed using the methodology shown in Table 1; the flakes were also converted into compression-molded film to determine their optical properties. The methodologies are described in detail in Supplementary Materials S1.

#### 2.3.3. Mass Balance and Calculation of the Washing Process

To assess the efficiency of the recycling process, the PET recycling yield (ηPET) for the 14 categories of PET trays was calculated according to Thoden van Velzen et al., 2017 [40]. Recovered PET material was calculated by applying Equation (1):

(1) $$\eta PET=\frac{c_{sinking\;fraction}^{PET}\cdot m_{sinking\;fraction}^{dm}}{c_{feedstock}^{PET}\cdot m_{feedstock}^{gross}\cdot nmc}$$

$c_{sinking\;fraction}^{PET}$ and $c_{feedstock}^{PET}$ represent the concentrations of PET in the sinking fraction and the feedstock;

$m_{sinking\;fraction}^{dm}$ is the dried weight of the sinking fraction;

$m_{feedstock}^{gross}$ is the dirty milled flakes (50 g);

*nmc* is the net material content.

#### 2.3.4. Recyclability Assessment Method

To assign the degree of recyclability to the different categories of PET trays, three levels of compatibility were distinguished.

**Figure 2:**
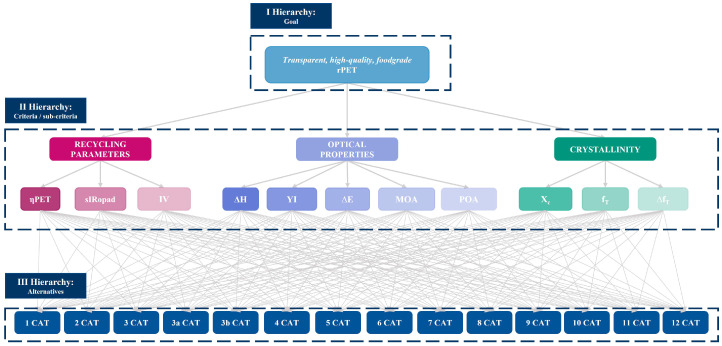
Structure of the multicriteria analysis from the 14 categories (CAT) of PET trays in a hierarchy. Note: ηPET = yield of the process; sIRopad = impurities % from sIRopad analysis; IV = intrinsic viscosity; ΔH = haze value compared with reference (mono PET tray); YI = Yellow Index value compared with reference (mono PET tray); ΔE = color (L*a*b) value compared with reference (mono PET tray); MOA = microscope optical analysis; POA = photo optical analysis; Xc = DSC crystallinity results; f_T_ = IR crystallinity results; Δf_T_ = crystallinity results.

A multicriteria decision analysis was carried out using the analytic hierarchy process (AHP), which was structured in three principal steps (see Figure 2). The first step establishes a hierarchical structure. At the first level of the hierarchy, the main objective is defined: to obtain transparent, high-quality and food-grade rPET. The relevant criteria and conditions are established in the middle step. The final step in the hierarchy involves identifying alternatives [19].

In this study, the criteria were related to the analyses performed at the steps of the mechanical recycling process. Indeed, three main criteria were identified: recycling parameters (RP), crystallinity (C) and optical properties (OP). The following evaluation sub-criteria were established:

- The yield (ŋPET) of the recycling process, in terms of recovered PET, could quantify the weight losses due to milling and alkaline hot washing, measured as described in Section 2.3.3.
- The content of impurities (sIRopad) detected by IR and sIRopad analyses in the final flake samples allows the definition of how well the standard recycling process removes some contaminants [40].
- The intrinsic viscosity (IV) of the recycled PET polymer relates to its molecular weight and determines its properties and applicability. Each mechanical treatment causes a decrease in IV value and, also, the high level of moisture and impurities leads to the reduction in rPET intrinsic viscosity during mechanical treatments [41], especially after thermal processes.
- The degree of crystallinity was determined by DSC (X_c_) and approximated with IR (f_T_, Δf_T_) analyses [42,43]. The crystallinity should be minimized to ensure transparency and clarity [44,45].
- The optical properties in terms of color (ΔE), yellowness (YI) and haze (ΔH) of the final obtained sheets and visually detected impurities (MOA: microscope optical analysis; POA: photo optical analysis). For PET packaging, optical properties, such as color and clarity, are key factors that may limit their applicability [5]. Also, the yellowing index contributes to determining the degree of degradation of rPET [46].

For each alternative, each category was assigned a score (pi,j) based on the analysis results (see Table S1.4). Therefore, each criterion and sub-criterion is analyzed individually to identify the related priority vectors (i.e., the weights assigned to each criterion and sub-criterion) [47]. The AHP uses the principal eigenvalue method to derive ratio-scale priority vectors from positive reciprocal matrices, which are established through pairs of comparisons [48]. To ensure the reliability of the weight assignment and minimize subjective bias, a consistency check was performed. In all stakeholder scenarios, the consistency ratio (CR) was below the 0.1 threshold, indicating that the comparisons were consistent and the derived weights were reliable for the recyclability assessment.

The weighting scheme for sub-criteria was defined based on the extent to which each parameter adds value to the main criterion to which it is assigned, as shown in Table 2a.

While weighing the main criteria, three scenarios based on stakeholders’ acceptance were explored to determine the correlated importance, a way of considering the social, technical, and legislative aspects. To minimize the subjectivity in weighting, a mixture of experts and public entities was selected: consumers, recyclers, and lawmakers. Weights were assigned to the comparison among the various categories based on the priority of pairs of criteria shown in Table 2b.

For consumers, the optical properties are most important because packaging aesthetics is the decisive factor in their purchasing decisions [49,50]. Consequently, color and haze emerge as paramount criteria for encouraging consumers to buy the packaged product. Instead, among the three, the recycler will prioritize the productivity aspects of the recycling process, i.e., primarily recycling yield and crystallinity over optical properties, ensuring greater final product yields. The legislator would insist on a harmonious balance of all characteristics, with a special emphasis on washing yield and optical properties, as they play a significant role in meeting the established recycling targets.

Once the weights are assigned, shown in Figure S1.3 (Tables S1.1 and S1.2), to each criterion (vi) and sub-criterion (wi,j), the final ranking has been assessed by calculating three times (one for each scenario) the global weights for each alternative with Equation (2):

(2) $${Gw}_{j}=\;\sum_{i=1}^{3}v_{i}\;\times \sum_{j=1}^{n}w_{i,j}\times \;p_{i,j}$$

where *j* varies from 1 to 14, as many as the number of PET tray categories;

*Gw_j_*, global weights related to each category;

*v_i_*, weight related to the three main criteria (Table S1.5);

*w_i,j_*, weight related to the different sub-criteria (Table S1.6);

*p_i,j_* corresponds to the score assigned to each sub-criteria based on the analysis values (Tables S1.3 and S1.4).

Therefore, based on the global weights obtained at the end of the MCDA for each scenario, three ranges of values were defined to indicate the level of compatibility with recycling (Table S1.7).

The various categories were associated with three levels of compatibility with the target set (recyclability levels) in each scenario. The categories with Gw ≥ 0.60 have the highest level of compatibility, with 0.50 ≥ Gw ≥ 0.60 having limited compatibility and Gw ≤ 0.50 having the lowest level of compatibility.

## 3. Results

### 3.1. Mechanical Recycling Process

**Figure 3:**
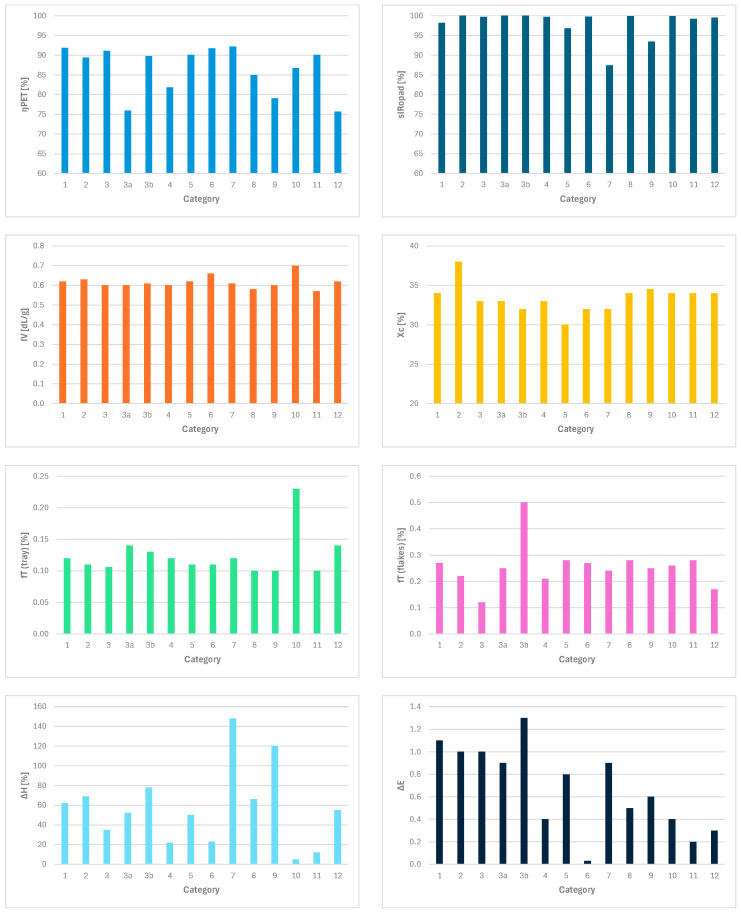

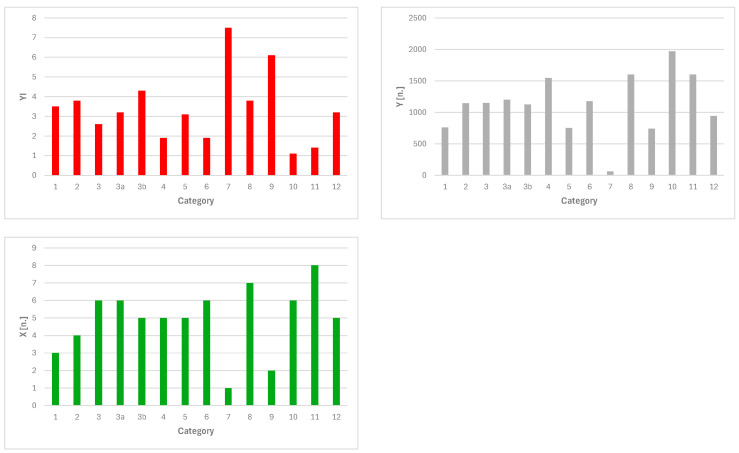
Overall results. PET tray categories: 1 = transparent meat, cheese, fish, pasta, vegetarian trays (multilayer); 2 = bowls and fresh salad trays; 3 = clamshells and top-sealed trays for fresh fruits, vegetables, and nuts; 3a = clamshells and top-sealed trays for fruit with PE bubble wrap inlay with hotmelt; 3b = clamshells and top-sealed trays with moisture absorber inlay with hotmelt; 4 = smearable salad trays; 5 = clear trays and clamshells for cookies and bakery products (mono PET); 6 = non-food blisters; 7 = thermoformed trays for cured meat products and sliced cheese; 8 = container for eggs; 9 = flat trays to support sliced meat and cheese products; 10 = jars (e.g., peanut butter, creams); 11 = loose lids and caps; 12 = pots for yogurt.

The thermal and optical analysis results are summarized in Table 3 and Figure 3 (detailed data are reported in Supplementary Materials S2). A detailed analysis of the results has been reported in the next sections.

#### 3.1.1. Recycling Yield

PET recycling yields ranged from 75% to 92% across the various types of PET trays. Some PET material is lost in the sludge as fines and during the sink–float separation. No clear relationship was found between this yield and the material composition of the PET trays (Table S2.1). The losses to fines are likely related to the thinness of the packaging types, which fragment into smaller pieces than PET bottles during shredding and washing [5]. Losses during sink–float separation are likely due to the complex composition of the packages, which contain PET and lower-density polymers such as PE and PP.

#### 3.1.2. Quality of the Recycled PET Made from Different PET Trays

Although the fourteen different types of PET trays can easily be recycled into washed flakes, only a few recycling products are composed of only PET flakes (type 2, type 3, and type 3a), and most also contain small amounts of other polymers/materials such as PE, PP, PS and cellulose (see Table S2.2). Most recycled PET products have total concentrations of polymeric contaminants below 1%. Only recycled PET from PET tray categories 5, 7 and 9 has much higher levels of polymeric contaminants, up to 13%. These polymeric contaminants originate from packaging components that cannot be separated during mechanical recycling. For example, the inner PE layer of a standard PET-PE meat tray (type 1) is coextruded on the PET and is only partially washed off and partially removed by sink–float separation. The recycled PET with the highest level of polymeric contamination was produced from thermoformed packages for sliced cured meat and cheese products (type 7). This package generally comprises a thermoformed tray, a top film and two labels, and all four components comprise multiple inseparable materials [7]. This also happens for type 5c, which has a similarly complex composition. For type 9 trays, which are generally mono-material, the feedstock was not only flat PET trays, but also multilayered flow-pack films composed of multiple polymers, such as PA, PET, PE and EVOH.

The IV values are relatively low compared to rPET from bottles [51] but agree with values from recycled PET trays that have not yet been subjected to SSP treatment (the IV of a reference mono A PET trays was determined to be 0.62 dL/g). These values correspond to molecular weights that vary between 28,000 and 35,000 g/mol [52] (Table S2.4). The category with the highest IV value is 10, which are injection-molded pots. It was expected that their IV would be larger than the IVs of the other categories, which are thermoformed trays.

The degree of crystallinity (X_c_) ranged from 30 to 38% across the various recycled PET products, and the fraction of glycol moieties in the trans configuration (f_T_) ranged from 12 to 50%. Hence, recycled PET products are semi-crystalline in general. Compared with the non-recycled trays, the fraction of glycol moieties in the trans configuration has increased from roughly 10 to 23% to 12 to 50%, indicating that the recycled PET flakes are slightly more crystalline than the PET packages from which they were produced.

**Figure 4:**
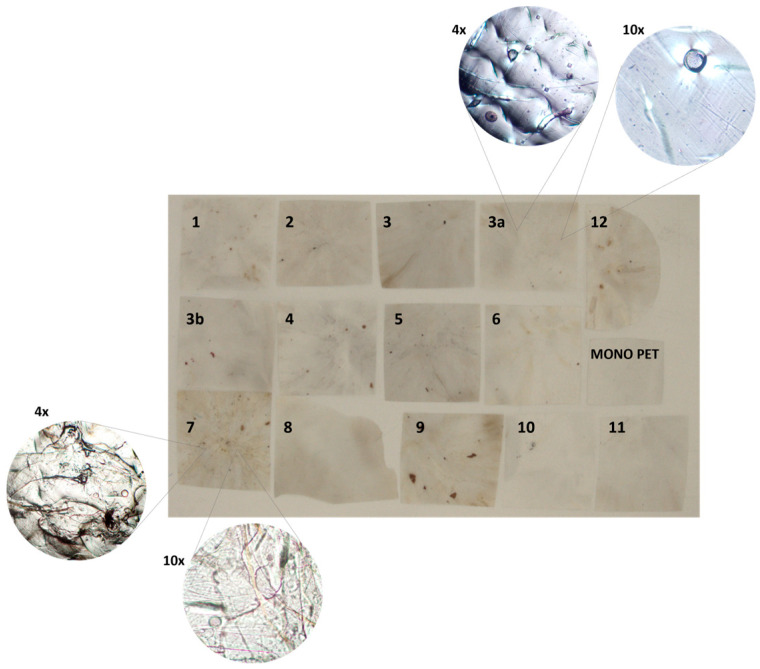
Photos of compression-molded rPET amorphous sheets per category.

The color of the recycled PET products is only a little bit more yellow than the color of the corresponding trays (see Table S2.7 and Figure 4). As the yellowing of PET indicates heat- and light-induced degradation reactions in the polymeric backbone [53,54], the limited yellowing of recycled PET indicates that the PET products have undergone little degradation. Furthermore, the recycled PET products are hazier than the PET packages from which they were produced, corresponding to high amounts of visible impurities and, hence, low scores for X and Y.

The high levels of impurities are partially due to polymeric contaminants in recycled PET products and to fibrous imperfections. When these recycled PET products are produced on larger scales and extruded with melt filtration, impurities are likely to be lower and more evenly dispersed. This would improve the optical properties of recycled PET products.

The mono A PET reference tray shows a* and b* values very close to zero, implying that it is uncolored. As shown in Figure 4, the compressed foils tend to have higher a* and YI values and are hence more yellow than the mono PET sample, especially for categories 7 and 9, as confirmed by the YI reported in Table S2.2.

Finally, optical microscopy images were examined to detect impurities and contaminants. The microscopic images and photos, presented as examples in Figure 4 (e.g., categories 3 and 7), were closely examined (Figures S1.1 and S1.2), confirming what was already evident from other analyses (e.g., color analyses). Specifically, category 7 exhibits significant contamination because of its multi-material composition, including various elements like colored films, labels and glue (Table S2.1). This contrasts sharply with category 3 images (mono-material), which display a more transparent appearance.

The presence of high levels of polymeric contaminants had a significant impact on the final quality of the rPET (particularly in categories 1, 5, and 7). High levels of impurities and residual moisture are known to cause a reduction in intrinsic viscosity (IV) during mechanical and thermal treatments, as they facilitate degradation reactions. Furthermore, the mechanical recycling process can induce chain scission of the polymer backbones, thereby increase crystallinity and reducing molecular weight. This structural alteration is particularly evident in categories with inseparable multi-material components (like the PET-PE layers in category 7), which result in polymeric blends with mediocre mechanical properties. These contaminants also act as precursors to thermal oxidation, as confirmed by increases in the Yellow Index (YI) and haze (ΔH), indicating that high concentrations of non-target polymers like PE, PP, or adhesives prevent the formation of a high-quality, transparent polymer matrix.

### 3.2. Recyclability Assessment

**Figure 5:**
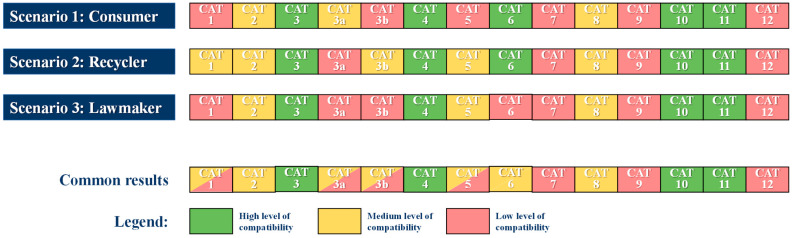
Final MCDA classification of the 14 categories into the three scenarios. PET tray categories: 1 = transparent meat, cheese, fish, pasta, vegetarian trays (multilayer); 2 = bowls and fresh salad trays; 3 = clamshells and top-sealed trays for fresh fruits, vegetables, and nuts; 3a = clamshells and top-sealed trays for fruit with PE bubble wrap inlay with hotmelt; 3b = clamshells and top-sealed trays with moisture absorber inlay with hotmelt; 4 = smearable salad trays; 5 = clear trays and clamshells for cookies and bakery products (mono PET); 6 = non-food blisters; 7 = thermoformed trays for cured meat products and sliced cheese; 8 = container for eggs; 9 = flat trays to support sliced meat and cheese products; 10 = jars (e.g., peanut butter, creams); 11 = loose lids and caps; 12 = pots for yogurt.

The MCDA engenders global weights as a ranking (Table S2.8) for each scenario and alternative. In each scenario, the alternative (PET tray type) with the highest global weight was deemed the best option (most recyclable) and the one with the lowest was deemed the worst. At the end of the multicriteria analysis, the 14 categories were ranked into three categories, one for each scenario (consumer, recycler, and lawmaker). A range of values was assigned (Table S1.7) to classify the categories into high, medium and low compatibility (Figure 5) to assess their recyclability.

Several observations can be made when the object and material compositions of the various PET tray categories (Figure 5) are compared to the PET yields and quality of the produced rPET.

Categories 1, 5, and 7 have the highest PE content in both the tray and the components, as well as a complex composition of components themselves: polypropylene, paper, metals and PSA (pressure-sensitive adhesives). Consequently, it is unsurprising that the MCDA classifies these PET trays as poorly recyclable (medium for category 5 in the second and third scenarios). Also, the 9th and 12th categories exhibit low compatibility across all scenarios, despite having low PE content. Particularly for category 9, this result may be related to the fragile nature of the thin trays themselves.

Categories 3, 4, 6, 10 and 11 are ranked highest in terms of recycling compatibility. Specifically, category 6 (non-food packaging blisters) produces a high-quality rPET and, therefore, receives the highest global weight in two scenarios (consumer and recycler points of view). However, this rPET is made from non-food packaging, and although it has been shown that food-grade rPET can be produced from non-food-PET packaging [55,56,57], EFSA requires that the feedstock be composed of 95% food packaging [58]. Therefore, despite this type of PET tray rendering a high quality of rPET, it is still awarded a low compatibility ranking in the legal scenario.

These categories of PET packages deliver high-quality rPET despite the complexity of their component composition; the trays are almost single-material, with negligible non-PET content. Thus, the high compatibility can be attributed to the efficient way the recycling process removes most unintended components (e.g., the PET packages in category 10 are closed with caps rather than a top-sealed film, and these caps are removed much more efficiently). In contrast, the second and eighth categories have medium compatibility, even though their composition is not particularly complex, reconfirming that it is not just the feedstock’s material composition that determines the quality of the rPET, but also the recycling process’s ability to separate the materials.

In general, the worst-performing categories are composed of multiple materials that a simple mechanical recycling process cannot separate, rendering polymeric blends with mediocre properties. The best-performing categories are composed of PET, and the other materials/components can be separated efficiently during recycling. Furthermore, common success factors among the best-performing categories for producing high-quality rPET include favorable degrees of crystallinity and relatively high levels of intrinsic viscosity of the PET material, as these contribute to the structural integrity and robustness of the packaging during milling and washing.

## 4. Discussion

**Figure 6:**
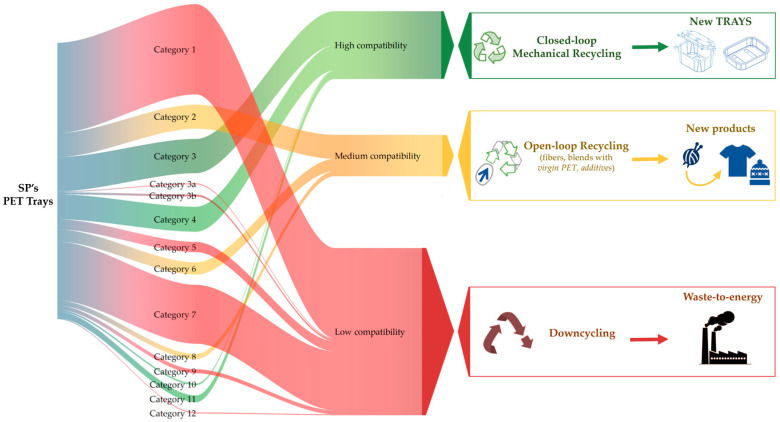
PET tray categories material flow. PET tray categories: 1 = transparent meat, cheese, fish, pasta, vegetarian trays (multilayer); 2 = bowls and fresh salad trays; 3 = clamshells and top-sealed trays for fresh fruits, vegetables, and nuts; 3a = clamshells and top-sealed trays for fruit with PE bubble wrap inlay with hotmelt; 3b = clamshells and top-sealed trays with moisture absorber inlay with hotmelt; 4 = smearable salad trays; 5 = clear trays and clamshells for cookies and bakery products (mono PET); 6 = non-food blisters; 7 = thermoformed trays for cured meat products and sliced cheese; 8 = container for eggs; 9 = flat trays to support sliced meat and cheese products; 10 = jars (e.g., peanut butter, creams); 11 = loose lids and caps; 12 = pots for yogurt.

The classification into three levels of compatibility with the applied mechanical process allows consideration of the End of Waste (EoW) pathways for the PET tray categories. In Figure 6, a hypothetical material flow based on the PET tray sorting data from the previous study [7] has been delineated based on the recyclability assessment, considering that:

- Categories 3, 4, 10, and 11 were found to be highly recyclable via mechanical recycling and suitable for closed-loop recycling.
- Categories 2, 6 and 8 were classified with medium compatibility for mechanical recycling; the EoW pathway involves open-loop recycling in rPET.
- The largest number of sorted products (as evident in Figure 6), comprising 7 of the 14 categories (1, 3a, 3b, 5, 7, 9, and 12), had the lowest mechanical recycling compatibility.

Categories with high compatibility could be included in a tray-to-tray system [59,60,61,62] where recycled raw material is used for the same product and fully replaces virgin material.

For categories resulting in medium compatibility, the open-loop recycling could be the proper recovery approach, involving the blending of a high proportion of virgin polymer with recycled PET [63] to manufacture new products or to make use of wide chain-extenders, heat stabilizers, processing aids (plasticizers), impact modifiers, fillers, crosslinkers or compatibilizers [64], to improve the overall mechanical properties of the resulting blend [65]. Indeed, compatibilization shows great promise as a possible solution to address mixed plastic waste [15,66,67,68,69].

Also, the recycling steps affect the physical properties of the recycled materials significantly, causing chain scission of the polymer backbones, increasing the crystallinity degree, reducing their molecular weight [70,71] and initiating hydrolytic degradation and thermal oxidation [44].

To address these issues, the solid-state polymerization (SSP) process has been shown to increase the molar mass of PET while slowing down the crystallization rate, but it has also demonstrated high effectiveness in decontaminating the material, making the influence of contaminants negligible [72,73].

For the stream of PET trays with low mechanical recyclability, the valorization path could involve depolymerization to generate chemicals for rPET or other commodities, which can be achieved via many different routes [34,74,75,76,77]. Dissolution-based plastic recycling approaches can have advantages over other recycling technologies, as plastics can be converted into fuels or monomers to produce the virgin resins. Chemical depolymerization is a solution for harder-to-recycle waste, such as colored PET, polyester fibers, multilayer tray waste, and waste from mechanical processes, while avoiding a mono-material stream [12]. Pyrolysis, liquefaction, and gasification have the advantage of processing mixtures of plastics and handling contamination more easily [78,79]. Also, there are still many limitations of the current techniques, such as economic investment [80] to realize the scaled applications, the strict operation conditions, the high cost and poor reusability of catalysts, the use of large amounts of solvent with related toxicity and energy consumption, and the scientific gaps to be addressed through the implementation of innovative technologies [81].

In addition, given the current technological state of real plants in Europe [82], it is probably more feasible to consider that this stream goes to incineration in the near future, also avoiding landfill as the worst EoW treatment for packaging waste [83], since incineration is by far the most common technology for Waste-to-Energy [84]. Nowadays, for multilayer packaging downcycling is seen as part of the solution, contributing to reducing plastic leakage into the environment [85,86].

In conclusion, by examining the types of categories and their composition, it is possible to identify two main streams of PET trays: one associated with medium- and high-compatibility categories, whose composition is less complex, and the other related to low-compatibility of multi-material PET tray streams, which can be recovered using secondary recovery technologies.

Nevertheless, the redesign of multi-material packaging, while ensuring the shelf life of packaged food products, results in the most sustainable solution for this waste stream [81,87,88]. Accordingly, if it had a simpler structure, it would entail higher rates of closed-loop production and higher rPET production rates. Despite the current European landscape presents an advanced configuration but still needs to be improved in terms of plants and techniques availability, as well as the packaging characteristics needing to be redesigned, a redistribution of mass flows can be expected in the future to allow closed-loop recycling of the entire stream of PET trays accomplished to satisfy the recycled PET demand.

## 5. Conclusions

The novel MCDA application assessed the recyclability of 14 types of PET trays into food-grade, transparent rPET from three perspectives: (i) social, (ii) technical, and (iii) legislative. Producing transparent, food-grade recycled PET from this feedstock through mechanical recycling remains highly challenging. PET trays are used to pack a range of items from food (meat, fish, cheese, hummus, salads, vegetables, yogurt, and creams) to non-food articles, which pose complexity due to varying material compositions. Some trays consist of seven or more components, incorporating different materials.

The 14 different types of PET trays were mechanically recycled, and the 14 different types of rPET produced were chemically and thermally analyzed. The following findings were achieved:

- About 72% of sorted products (7 of the 14 categories) were recoverable through chemical or thermal recycling, which are considered secondary recycling processes, as they yield lower-value recovery compared to primary recycling, which maintains material quality and enables true closed-loop recycling.
- Only around 28% of the sorted products (from the remaining 7 categories) resulted in enabling mechanical recycling.

The results showed that recyclability is not solely determined by the material composition of the feedstocks; other factors are also important. Namely, the package design is relevant because it determines the likelihood of separation into various components and materials during the recycling process. Finally, the robustness of the PET package is also relevant, as a PET tray with optimal thickness, degree of crystallinity, and high IV values will not fragment easily during shredding and washing and will not produce fines that are lost to the sludge.

This study was conducted at the laboratory bench scale; therefore, future research should verify these results in industrial-scale recycling processes to account for the complexities of large-scale melt filtration and continuous processing. Additionally, subsequent work may explore the application of compatibilizers to enhance the mechanical performance of multi-material blends or the use of solid-state polymerization (SSP) to restore the molecular weight and intrinsic viscosity of rPET from trays. These advancements, combined with a focus on eco-design to simplify packaging structures, will be essential for achieving a truly circular economy for non-bottle PET waste.

## Figures and Tables

**Table 1:** Methods for characterization of rPET flakes and sheets.

Method	Instrument	Parameter	Reference
Infrared (IR) spectroscopy analysis	IoSys-SIROpad NIR analyzer (IOSYS GmbH, Steinhauserstrasse 14, Ratingen, Germany)	PET content in rPET flakes $c_{sinkingfraction}^{PET}$	[27]
	Bruker Alpha Fourier Transform IR (FTIR) (Bruker Nederland BV, Elisabethhof 15, Leiderdorp, The Netherlands)	Fraction of glycol moieties in the trans configuration $f_{T}$	[28,29,30,31,32]
Differential scanning calorimetry (DSC) analysis	Perkin-Elmer DSC-8000 calorimeter (Perkin-Elmer Nederland BV, Nieuwe Langeweg 41, Hoogvliet, The Netherlands)	Crystallinity degree $Xc\left(wt.\%\right)$	[33,34]
Intrinsic Viscosity (IV) measurement	Schott Geräte CT1250 Schott Geräte AVS350 (Schott Benelux BV, Randweg 3A, Rotterdam, The Netherlands)	Intrinsic viscosity η Molecular weight $M_{w}$	[35,36]
Microscope optical analysis (MOA)	Konica Minolta Chroma meter CR—5 (Konica Minolta business solutions BV, Capellalaan 65, Hoofddorp, The Netherlands)	Total color difference ΔE Yellowness index YI	[37,38]
Photos optical analysis (POA)	BRESSER Science MPO 401 Microscope MikroCam II 20 MP 1 “Lightening cabinet” (Bresser GmbH, Gutenbergstrasse 2, Rhede, Germany)	Visible impurities (counts X and Y)	[39]

**Table 2:** (**a**) Order of priority of the evaluation sub-criteria. (**b**) Stakeholder scenarios assumed about criteria priorities.

(**a**)	**SUB-CRITERIA**		
	Criteria	ID Criteria	Order of Priority of the Evaluation Sub-Criteria
	Recycling Parameters	RP	ηPET > sIRopad > IV
	Optical Properties	OP	ΔH > YI = ΔE > MOA > POA
	Crystallinity	C	DSC > f_T_ > Δf_T_
(**b**)	**STAKEHOLDER SCENARIOS**		
	Stakeholder Scenario	ID Scenario	Order of Priority of the Evaluation Criteria
	Consumer	Scenario 1	OP > RP > C
	Recycler	Scenario 2	RP > C > OP
	Lawmaker	Scenario 3	RP = OP > C

**Table 3:** Overall results.

CAT	ŋPET [%]	sIRopad [%]	IV [dL/g]	Xc [%]	fT (Tray) [%]	fT (Flakes) [%]	∆H [%]	∆E [-]	YI [-]	Y [n.]	X [n.]
**1**	91.84	98.2	0.62	34	0.12	0.27	62	1.1	3.5	760	3
**2**	89.40	100	0.63	38	0.11	0.22	69	1.0	3.8	1147	4
**3**	89.74	99.72	0.60	33	0.106	0.12	35	1.0	2.6	1148	6
**3a**	75.97	100	0.60	33	0.14	0.25	52	0.9	3.2	1200	6
**3b**	91.08	100	0.61	32	0.13	0.50	78	1.3	4.3	1127	5
**4**	81.85	99.7	0.60	33	0.12	0.21	22	0.4	1.9	1544	5
**5**	90.08	96.8	0.62	30	0.11	0.28	50	0.8	3.1	753	5
**6**	91.74	99.8	0.66	32	0.11	0.27	23	0.03	1.9	1176	6
**7**	92.21	87.4	0.61	32	0.12	0.24	148	0.9	7.5	62	1
**8**	84.95	99.998	0.58	34	0.10	0.28	66	0.5	3.8	1600	7
**9**	79.07	93.44	0.60	34.5	0.10	0.25	120	0.6	6.1	740	2
**10**	86.74	99.9	0.70	34	0.23	0.26	5	0.4	1.1	1970	6
**11**	90.07	99.2	0.57	34	0.10	0.28	12	0.2	1.4	1600	8
**12**	75.70	99.5	0.62	34	0.14	0.17	55	0.3	3.2	945	5

PET tray categories (CAT): 1 = transparent meat, cheese, fish, pasta, vegetarian trays (multilayer); 2 = bowls and fresh salad trays; 3 = clamshells and top-sealed trays for fresh fruits, vegetables, and nuts; 3a = clamshells and top-sealed trays for fruit with PE bubble wrap inlay with hotmelt; 3b = clamshells and top-sealed trays with moisture absorber inlay with hotmelt; 4 = smearable salad trays; 5 = clear trays and clamshells for cookies and bakery products (mono PET); 6 = non-food blisters; 7 = thermoformed trays for cured meat products and sliced cheese; 8 = container for eggs; 9 = flat trays to support sliced meat and cheese products; 10 = jars (e.g., peanut butter, creams); 11 = loose lids and caps; 12 = pots for yogurt.

## Data Availability

The original contributions presented in this study are included in the article and Supplementary Materials. Further inquiries can be directed to the corresponding authors.

## Supplementary Materials

The following supporting information can be downloaded at: https://www.mdpi.com/article/10.3390/polym18091063/s1, Supplementary Material S1: Figure S1.1: Square grid for visual counting on photos of compression-molded rPET sheets for categories; Figure S1.2: Example of square grid for visual counting on microscope images of compression-molded rPET sheets; Figure S1.3: Assigned weights for criteria and sub-criteria for the 3 scenarios; Table S1.1: Average material composition per PET tray category (PSA = pressure-sensitive adhesive); Table S1.1: Selected assessment sub-criteria for comparing PET tray categories; Table S1.2: Score determination for sub-criteria; Table S1.3: Range values for scoring; Table S1.4: Categories scoring for criteria—pi,j; Table S1.5: Weights for main criteria for scenario—vi; Table S1.6: Weights for sub-criteria (the same for all scenarios)—wi,j; Table S1.7: Score for the three levels of compatibility to recycling. Supplementary Material S2: Table S2.1: PET tray category composition; Table S2.2: Sinking fraction composition per category; Table S2.3: Yields of laboratory-scale recycling per category; Table S2.4: Intrinsic viscosity and molecular weight values of PET flakes after washing per category; Figure S2.1: Thermogram of DSC measurements of a sample of the 12th category; Table S2.5: DSC data for category: melting temperature, onset of cooling temperature, degree of crystallinity values and glass transition temperature; Figure S2.2: Spectra comparison between tray and flake samples wavelength 1320–1520 cm^−1^ of a sample of the first category; Table S2.6: Values of fT detected from IR spectra for tray and flakes samples of the 14 PET tray categories; Table S2.7: Results of L*a*b* and haze values referred to mono A PET tray (Bliston) of compression-molded foil per category; Figure S2.3: Results of L*a*b* and haze values referred to mono A PET tray (Bliston) of compression-molded foil per category; Figure S2.4: (a) Average material composition of total package for each category [% m/m]; (b) average material composition of components for each category [% m/m]. Table S2.8: Global weights for recycling assessment of the 14 categories. References [89,90,91,92,93,94,95,96,97] are cited in the Supplementary Materials.

## Abbreviations

AHP—Analytic hierarchy process; ATR-FT-IR—Attenuated total reflection Fourier transform infrared; C—Crystallinity; DKR—Deutsche Kreislauf und Recycling Gesellschaft; DSC—Differential scanning calorimetry; EFSA—European Food Safety Authority; EoW—End-of-Waste; EVOH—Poly(ethylene-co-vinyl alcohol); IR—Infrared; IV—Intrinsic viscosity; LDPE—Low density polyethylene; MCDA—Multi-criteria decision analysis; MOA—Microscope optical analysis; NIR—Near InfraRed; OP—Optical properties; PE—Polyethylene; PET—Polyethylene terephthalate; POA—Photos optical analysis; PP—Polypropylene; PS—Polystyrene; PVC—Polyvinyl chloride; RP—Recycling parameters; rPET—Recycled polyethylene terephthalate; SP—Sorted products; SWM—Solid waste management; SSP—Solid-state polycondensation; YI—Yellow Index.
