# Supplementary material for: Multi-Criteria Decision Analysis for Mechanical Recyclability Assessment of Different Types of PET Packaging Waste
Source: Polymers (Basel). 2026 Apr 28;18(9):1063. doi: 10.3390/polym18091063 (PMC13166004; doi:10.3390/polym18091063)
Supplement: Supplementary file 1 [file polymers-18-01063-s001.zip › Supplementary materials 1.pdf]

polymers

MDPI

Article – Supplementary Material 1: Methods

Multi-Criteria Decision Analysis for mechanical recyclability assessment of different types of PET packaging waste

Giusy Santomasi[1], Francesco Todaro[1,*] , Michele Notarnicola[1], Eggo Ulphard Thoden van Velzen[2,*]

[1] Department of Civil, Environmental, Land, Building Engineering and Chemistry (DICATECh), Polytechnic University of Bari, Via E. Orabona n.4, I-70125 Bari, Italy
[2] Wageningen Food & Biobased Research, Wageningen University & Research, Bornse Weilanden 9, 6709 WG Wageningen, the Netherlands

* Correspondance: francesco.todaro@poliba.it, FT ; ulphard.thodenvanvelzen@wur.nl; EUTvV

1. Methods

1.1 Characterization of rPET

1.1.1 sIRopad analysis

The material composition of the produced sinking fraction (30 - 40 g per category) was determined with a NIR Analyser (IOSYS-SIROpad, IOSYS, Germany) [1] through a Near-Infrared measuring system for plastic flakes and granules (non-black), allowing to measure the concentration of PET in the recovered product ($c^{PET}_{sinking fraction}$). Samples of about 10 grams were weighed and evenly distributed on the reflection plate, assuring that all flakes are separately located on the surface without overlap, to be scanned and identified individually. All flakes from a different polymer type are pin-pointed and manually removed. The flakes recognised as the same polymer were weighed on the analytical balance. These weights were used to calculate each polymer fraction's mass-based polymeric compositions and percentage composition.

1.1.2 ATR-FTIR spectroscopy

Infrared spectroscopy is a valuable tool for studying molecular structure, and it has been widely used for PET. In this study, the IR technique was used to define the differences between samples of the PET trays before recycling and of the washed flakes after recycling. IR conformational bands were used to approximate the fraction of glycolic segment in trans conformation (f_T), which is related to the fraction of amorphous phase according to [2]:

$$f_T = \frac{A_{1340}}{A_{1340}+kA_{1370}} \qquad \text{(Eq. S1.1)}$$

In Eq. S1.1, Ai is the integrated absorption intensity of the infrared absorption bands that have a maximum at wave number i (where = 1340 or 1370), and k is a constant derived by Bertoldo for a free-standing reference PET film of 20 mm thickness and is equal to 6.7.

The washed flakes were also analysed with a Fourier Transform IR (FTIR) Instrument (Alpha, Bruker Optics, Germany) equipped with a platinum ATR single reflection diamond sampling module. Data points were collected between the wavenumber range of 400 – 4000 cm-1 at a resolution of 4 cm-1 [3], controlled by Optics User Software (OPUS)

version 8.1. Spectra analyses were determined using OPUS version 8.1. (Kumagai et al., 2002; Masoumi & Safavi, 2012; Rani et al., 2019).

1.1.3 Differential Scanning Calorimetry (DSC)

A calorimeter (DSC-8000, Perkin-Elmer, USA) was used to obtain thermograms of 10-15 mg of samples of PET tray from the different categories. The closed cups with samples were subjected to the following program: conditioning for 5 minutes at 0°C, heating at 10°C per minute to 300°C, cooling to 0°C, conditioning for 5 minutes at 0°C, heating at 10°C per minute to 300°C. Two samples were measured from each PET tray category. The following parameters were derived from the recorded thermograms: glass transition temperature (Tg), crystallisation temperature (Tc), melting temperature (Tm), enthalpy of crystallisation (ΔHc), and enthalpy of melting (ΔHm). Melting peaks and glass transition temperatures tentatively identified the polymers that are present in the samples [4].

The melting point and the melting temperature were derived from the second heating curve, assuming a theoretical melt enthalpy for a PET crystal of 135.8 J/g. The degree of crystallinity was determined from the melting peak and the cold crystallisation peak according to the method of Torres et al. [5]:

$$Xc(wt.\%) = 100\frac{\Delta \mathrm{H}_m\,(T_m) - |\Delta \mathrm{H}_c(T_c)|}{\Delta \mathrm{H}_m^0(\mathrm{T}_m^0)} \qquad \text{(Eq. S1.2)}$$

where Xc is the degree of crystallinity, ΔHm (Tm) is the enthalpy of fusion measured at the melting point, Tm, and $\Delta \mathrm{H}_m^0$ (T_m^0) is the enthalpy of fusion of the crystalline is invariably taken as the value at the equilibrium melting point) polymer measured at the equilibrium melting point, T_m^0.

1.1.4 Intrinsic viscosity measurement

The intrinsic viscosity (IV) was determined according to ASTM D4603-03 [6]. Samples of 250 mg PET flakes were dissolved in 50 mL phenol/tetrachloroethane (60/40 w/w) at 100°C for 30 minutes. After preparing the solution, the viscosities were determined in calibrated Cannon-Fenske capillaries of type 100 (as well as a reference solvent) at 30°C. The set-up included a water bath (CT1250, Schott Geräte, Germany), a thermocontroller, and a water cooler (CK100). The electronic measurements were done with a Schott Geräte AVS350. The intrinsic viscosity (η) was calculated by using the Billmeyer [7] relationship:

$$\eta = \frac{0.25\,(\eta_r - 1 + 3\ln\eta_r)}{C} \qquad \text{(Eq. S1.3)}$$

Where C is the concentration of the PET solution; η_r the relative viscosity of the solution (the ratio of the flow time of the solution to the flow time of the pure solvent).

Two samples per type were analysed; the results are presented as the average and standard deviations of the two measurements.

Subsequently, the viscosity-average molecular weight (Mw) of the samples could be calculated based on the viscosity data with the following expression [8]:

$$M_w = (\frac{\eta}{K})^{\frac{1}{\alpha}} \qquad \text{(Eq. S1.4)}$$

Where K= 7.44 * 10-4 and α= 0.648.

1.1.5 Color and haze measurements

The flakes were first converted into compression molded film to determine the optical properties of recycled PET products. A sample of 5-10 grams of washed flakes was compressed in a heated hydraulic press (PHI, USA) [9]. The press plates were heated at 280 °C for 5 minutes to allow the melting of all the flakes; applying minimum pressure,

the samples were pressed between two steel plates separated by Teflon sheets. Cooling was performed as fast as possible in iced water at 0 °C to avoid PET crystallization and to obtain amorphous PET foils of about 20 cm in diameter and less than 1 mm thick. Then, these PET foils have been studied through optical analysis.

Color was measured using a Konica Minolta Chroma meter CR-5 according to the CIEL*a*b* method [10,11] on the compressed molded films of each type of recycled PET. A white tile was used as a background reference. This yielded three color parameters: L*(100 = white; 0 = black), a* (positive = red; negative = green; 0 = grey), and b* (positive = yellow; negative = blue; 0 = grey). The total color difference ΔE and the yellowness (YI) of the samples were calculated as:

$$\Delta E = [(\Delta L^*)2+(\Delta a^*)2+(\Delta b^*)2]1/2 \qquad \text{(Eq. S1.5)}$$

indeed, the yellow index (YI) was calculated according to the equation as showed by Boehme M. et al. (2005) [12] and reported here:

$$YI= 142.86*b^*/L^* \quad \text{(Eq. S1.6)}$$

Haze measurements were simultaneously determined as the colour measurement on the same samples and with the Chroma meter (CR-5, Konica Minolta, Japan). Four haze measurements were recorded and then averaged for each sample, yielding average CIELab values for haze, L*, a* and b* and their standard deviations. Then, the total colour difference, yellowing and haze, was determined between the rPET sheets and mono-PET tray products, used as a standard reference. Also, photos were taken of the PET sheets in the lighting cabinet under controlled (fixed) lighting conditions.

1.1.5 Microscope analysis

Optical studies were performed on obtained rPET sheets utilizing Microscope (Science MPO 401, Bresser, Germany) [13]. It comprises 2 eyepieces, a 360° rotatable mechanical stage, and a center-adjustable and full rotatable object stage with a degree scale to change the object's direction in a controlled way. The illumination and observation unit encloses, e.g. the halogen bulb lamp (20 W) with dimmer, the field diaphragm, the 360° rotatable polarizer, centering implements for the objectives, and the analyzer attachment. Also, the microscope is equipped with an objective lens having magnification from 40 to 1000. For the analysis of PET foils, 4x and 10x magnifying camera lenses were employed to obtain well-defined images, and to take the photos, the Microscope Camera (MikroCam II 20 MP 1”, Bresser, Germany) was used.

The photos from the lighting cabinet and the images from the microscope were analyzed for visible imperfections. These imperfections were counted by creating a square grid on the images and counting the squares affected by the visible light, yellow, dark impurities, wires, and drops concerning transparent squares. This gave two values: named X, quantifying the squares in the grid that are not affected by coarse impurities in the regular photo (Figure S1.1), and Y, quantifying the squares in the grid that are not affected by micro impurities on microscope images (Figure S1.2). Lower values of these factors correspond to low optical quality, thus increased impurities content.

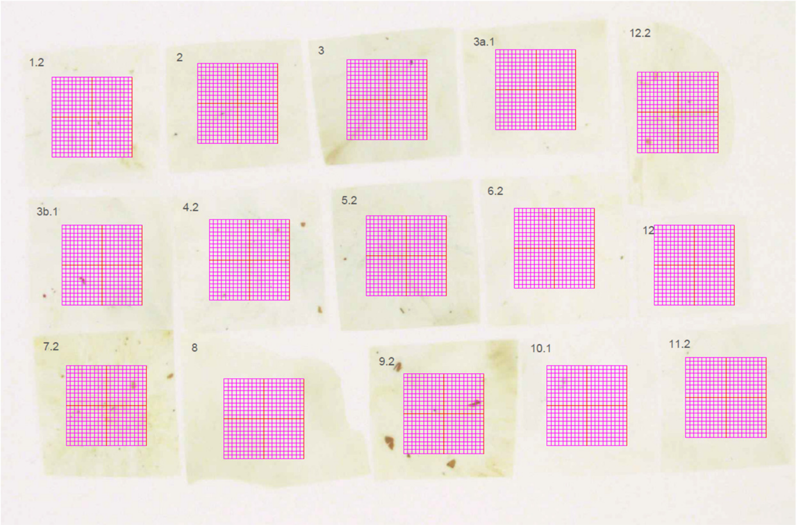

Figure S1.1. Square grid for visual counting on photos of compression moulded rPET sheets for categories.

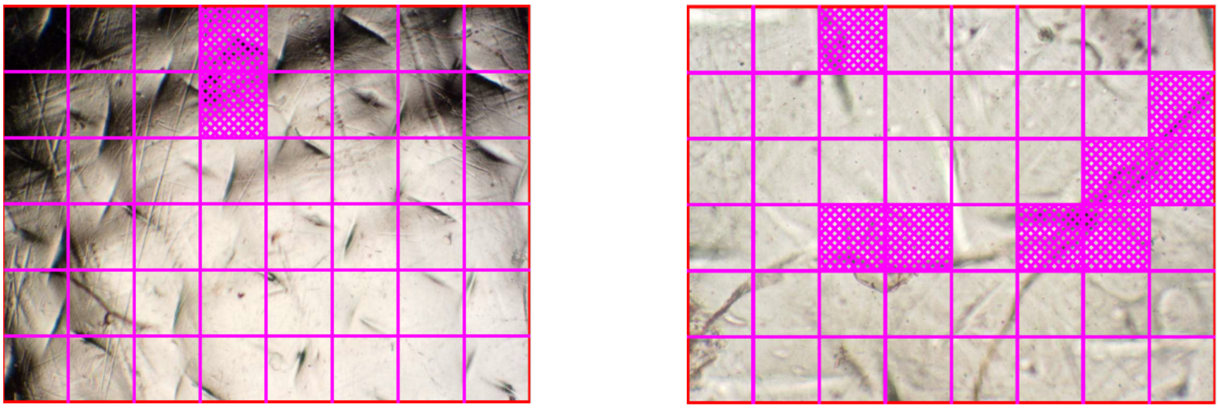

a)

b)

Figure S1.2. Example of square grid for visual counting on microscope images of compression moulded rPET sheets: a) magnitude 4x and b) magnitude 10x.

1.2 Multi-Criteria Decision Analysis

Table S1.1. Selected assessment sub-criteria for comparing PET tray categories.

Sub-criteria description		
RECYCLING PARAMETERS	**ηPET**	Yield of the process
	**sIRopad**	Impurities from sIRopad analysis
	**IV**	Intrinsic viscosity
OPTICAL PROPERTIES	**ΔH**	haze value compared with reference (mono PET tray)
	**YI**	Yellow Index value compared with reference (mono PET tray)
	**ΔE**	Colour (L*a*b) value compared with reference (mono PET tray)
	**MOA**	Microscope optical analysis
	**POA**	Photos optical analysis
CRYSTALLINITY	**X_c (%)**	DSC crystallinity results
	**Δf_T**	IR crystallinity results
	**f_T**	IR crystallinity results compared with reference (mono PET tray)

Table S1.2. Score determination for sub-criteria.

Range values for scoring sub-criteria				
**RECYCLING PARAMETERS**	**ηPET**	$\eta \geq 90\%$	$80\% < \eta < 90\%$	$\eta \leq 80\%$
	**sIRo-pad**	PET ≥ 99.99%	95% < %PET < 99.99%	%PET < 95%
	**IV**	$IV > 0.65$	$0.60 \leq IV \leq 0.65$	$IV < 0.60$
**OPTICAL PROPERTIES**	**ΔH**	$\Delta H < 40$	$25 \leq \Delta H \leq 70$	$\Delta H > 70$
	**YI**	ΔYI < 1	1 ≤ ΔYI ≤ 2.5	ΔYI > 2.5
	**ΔE**	$\Delta E < 1$	$1 \leq \Delta E \leq 2.5$	$\Delta E > 2.5$
	**MOA**	X > 5	3 ≤ X ≤ 5	X < 3
	**POA**	$Y \geq 1500$	$1500 > Y > 1000$	$Y \leq 1000$
**CRYSTALLINITY**	**X_c (%)**	$X_c < 32$ %	32 % ≤ X_c ≤ 35 %	$X_c > 35$ %
	**Δf_T**	$\Delta f_T \leq 0.11$	$0.11 < \Delta f_T < 0.15$	$\Delta f_T \geq 0.15$
	**f_T**	$f_T \leq 0.11$	$0.11 < f_T < 0.15$	$f_T \geq 0.15$
SCORE ($p_{i,j}$)		0.75	0.50	0.25

Table S1.3. Range values for scoring. Legend: bad = 0.25; medium = 0.50; good = 0.75.

	η^{PET}	**sIRopad**	**IV**	**ΔH**	**ΔE**	**POA**	**MOA**	**YI**	**f_T**	**Δf_T**	**X_c**
**High value**	0.75	0.75	0.75	0.25	0.25	0.75	0.75	0.25	0.25	0.25	0.25
**Medium value**	0.5	0.5	0.5	0.5	0.5	0.5	0.5	0.5	0.5	0.5	0.5
**Low value**	0.25	0.25	0.25	0.75	0.75	0.25	0.25	0.75	0.75	0.75	0.75

Table S1.4. Categories scoring for criteria – $p_{i,j}$.

	η^{PET}	sIRopad	IV	ΔH	ΔE	POA	MOA	YI	fT	ΔfT	Xc
**Alternative 1**	0.50	0.50	0.50	0.50	0.50	0.25	0.25	0.50	0.50	0.25	0.50
**Alternative 2**	0.50	0.75	0.50	0.50	0.50	0.5	0.50	0.50	0.75	0.50	0.25
**Alternative 3**	0.50	0.75	0.50	0.75	0.50	0.5	0.75	0.50	0.75	0.75	0.50
**Alternative 3a**	0.25	0.75	0.50	0.50	0.50	0.5	0.75	0.50	0.50	0.50	0.50
**Alternative 3b**	0.75	0.75	0.50	0.25	0.25	0.5	0.50	0.25	0.50	0.25	0.50
**Alternative 4**	0.50	0.75	0.50	0.75	0.75	0.75	0.50	0.75	0.50	0.75	0.50
**Alternative 5**	0.50	0.50	0.50	0.50	0.50	0.25	0.50	0.50	0.75	0.25	0.75
**Alternative 6**	0.75	0.75	0.75	0.75	0.75	0.5	0.75	0.50	0.75	0.25	0.75
**Alternative 7**	0.75	0.25	0.50	0.25	0.25	0.25	0.25	0.25	0.50	0.50	0.75
**Alternative 8**	0.50	0.75	0.25	0.50	0.50	0.75	0.75	0.50	0.75	0.25	0.50
**Alternative 9**	0.25	0.50	0.50	0.25	0.25	0.25	0.25	0.25	0.75	0.25	0.50
**Alternative 10**	0.50	0.75	0.75	0.75	0.75	0.75	0.75	0.75	0.25	0.75	0.50
**Alternative 11**	0.75	0.75	0.25	0.75	0.75	0.75	0.75	0.75	0.75	0.25	0.50
**Alternative 12**	0.25	0.75	0.50	0.50	0.50	0.25	0.50	0.50	0.50	0.75	0.50

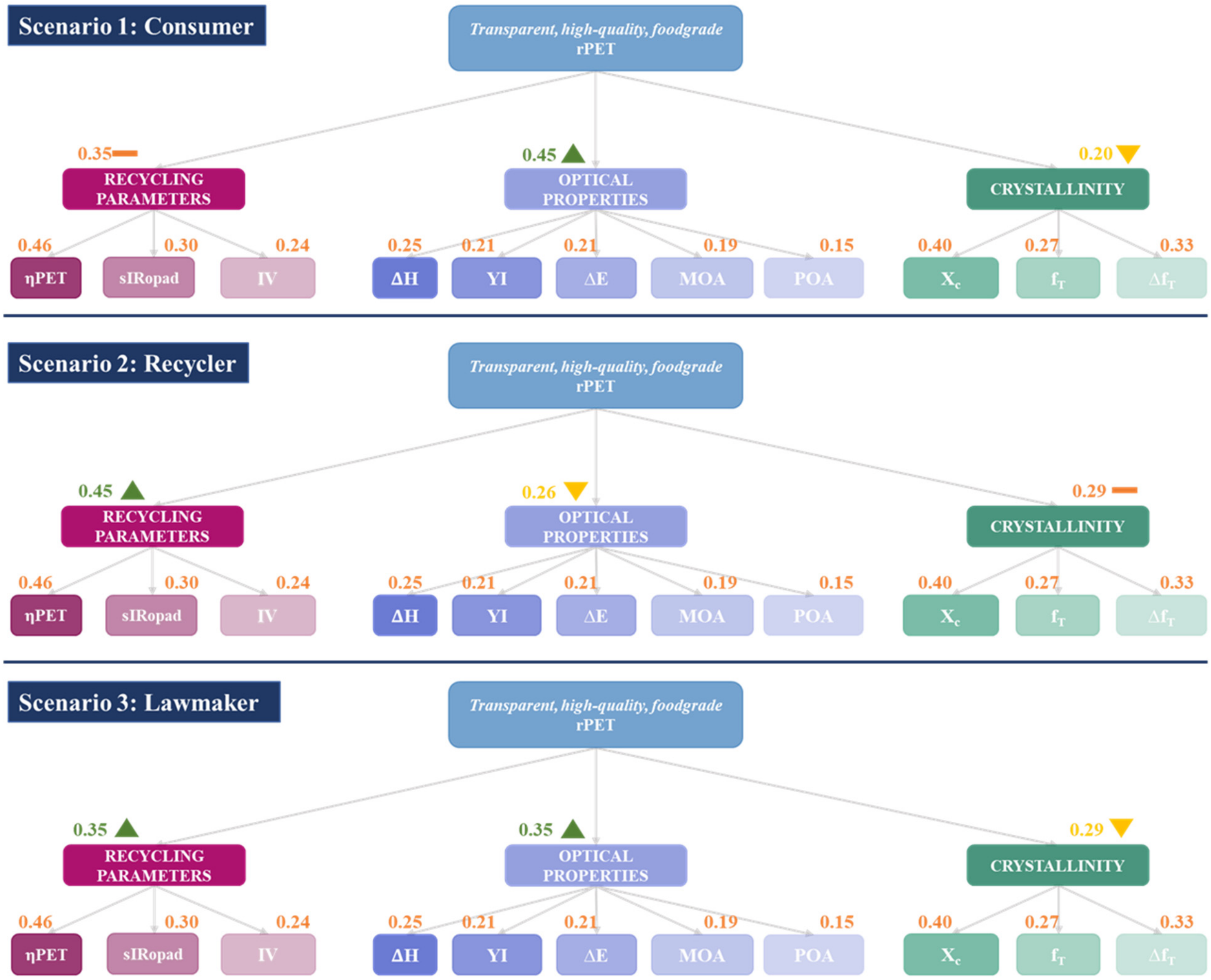

Figure S1.3. Assigned weights for criteria and sub-criteria for the 3 scenarios.

Table S1.5. Weights for main criteria for scenario – v_i.

	Scenario 1	Scenario 2	Scenario 3
**Recycling parameters**	0.35	0.45	0.35
**Optical properties**	0.45	0.26	0.35
**Crystallinity**	0.20	0.29	0.29

Table S1.6. Weights for sub-criteria (the same for all scenarios) - $w_{i,j}$.

Sub-criteria	Weights
**ηPET**	0.46
**sIRopad**	0.30
**IV**	0.24
**ΔH**	0.25
**YI**	0.21
**ΔE**	0.21
**MOA**	0.19
**POA**	0.15
**X_c**	0.40
**f_T**	0.27
**Δf_T**	0.33

Table S1.7. Score for the three levels of compatibility to recycling.

Level of recyclability	Score
High	≥ 0.60
Medium	0.50 - 0.60
Low	≤ 0.50

References

1. Alvarado Chacon, F.; Brouwer, M.T.; Thoden van Velzen, E.U.; Smeding, I.W. A First Assessment of the Impact of Impurities in PP and PE Recycled Plastics; **2020**, doi:10.18174/518299.
2. Bertoldo, M.; Labardi, M.; Rotella, C.; Capaccioli, S. Enhanced Crystallization Kinetics in Poly(Ethylene Terephthalate) Thin Films Evidenced by Infrared Spectroscopy. *Polymer (Guildf)* **2010**, *51*, 3660–3668, doi:10.1016/j.polymer.2010.05.040.
3. Anja Mieth; Eddo Hoekstra; Catherine Simoneau Guidance for the Identification of Polymers in Multilayer Films Used in Food Contact Materials: User Guide of Selected Practices to Determine the Nature of Layers. *JRC Science Hub* **2016**, doi:doi:10.2788/10593.
4. He, J.J.; Gilpatrick, B. Applications of DSC in conjunction with FTIR in plastic identification; **1999**;
5. Torres, N.; Robin, J.J.; Boutevin, B.; Re, C.E.; Ma, P. Study of Thermal and Mechanical Properties of Virgin and Recycled Poly(Ethylene Terephthalate) before and after Injection Molding; **1999**;
6. Hosseini, S.S.; Taheri, S.; Zadhoush, A.; Mehrabani-Zeinabad, A. Hydrolytic Degradation of Poly(Ethylene Terephthalate). *J Appl Polym Sci* **2007**, *103*, 2304–2309, doi:10.1002/app.24142.
7. Billmeyer, F.W. Methods for Estimating Intrinsic Viscosity; **1949**;
8. Berkowitz, S. Viscosity-Molecular Weight Relationships for Poly(Ethy1ene Terephthalate) in Hexafluoroisopropanol-Pentafluorophenol Using SEC-LALLS; **1984**;
9. Adebayo, G.O.; Yahya, R. Characterisation of Heat Modified Mangrove Fibre for Polymer Composite Applications; **2017**;
10. Arrieta, M.P.; López, J.; Ferrándiz, S.; Peltzer, M.A. Characterization of PLA-Limonene Blends for Food Packaging Applications. *Polym Test* **2013**, *32*, 760–768, doi:10.1016/J.POLYMERTESTING.2013.03.016.
11. Saberi, B.; Thakur, R.; Vuong, Q. V.; Chockchaisawasdee, S.; Golding, J.B.; Scarlett, C.J.; Stathopoulos, C.E. Optimization of Physical and Optical Properties of Biodegradable Edible Films Based on Pea Starch and Guar Gum. *Ind Crops Prod* **2016**, *86*, 342–352, doi:10.1016/j.indcrop.2016.04.015.
12. Boehme, M.; Charton, C. Properties of ITO on PET Film in Dependence on the Coating Conditions and Thermal Processing. *Surf Coat Technol* **2005**, *200*, 932–935, doi:10.1016/j.surfcoat.2005.02.040.
