# Supplementary material for: Multi-Criteria Decision Analysis for Mechanical Recyclability Assessment of Different Types of PET Packaging Waste
Source: Polymers (Basel). 2026 Apr 28;18(9):1063. doi: 10.3390/polym18091063 (PMC13166004; doi:10.3390/polym18091063)
Supplement: Supplementary file 1 [file polymers-18-01063-s001.zip › Supplementary materials 2.pdf]

polymers

MDPI

Article – Supplementary Material 2: Data

Multi-Criteria Decision Analysis for mechanical recyclability assessment of different types of PET packaging waste

Giusy Santomasi[1], Francesco Todaro[1] , Michele Notarnicola[1], Eggo Ulphard Thoden van Velzen[2,*]

[1] Department of Civil, Environmental, Land, Building Engineering and Chemistry (DICATECh), Polytechnic University of Bari, Via E. Orabona n.4, I-70125 Bari, Italy
[2] Wageningen Food & Biobased Research, Wageningen University & Research, Bornse Weilanden 9, 6709 WG Wageningen, the Netherlands

* Correspondence: francesco.todaro@poliba.it, FT ; ulphard.thodenvanvelzen@wur.nl; EUTvV

1. Data

Table S2.1. PET tray category composition [1].

Note: the observed variance in Tables S2.1 is scientifically justified by the high intrinsic heterogeneity of the lightweight packaging (LWP) waste stream and by the adopted sampling methodology. The data are derived from the average of waste samples and complete packaging samples to approximate a 50 g feedstock, which inherently contributes to variability.

	Material composition						
	**PET [%]**	**PE [%]**	**PP [%]**	**Other plastic [%]**	**Paper [%]**	**Metal [%]**	**PSA [%]**
**1**	73.0 ± 42.1	15.6 ± 8.4	1.3 ± 2.1	0.1 ± 0.6	8.3 ± 15.7	-	1.7 ± 2.8
**2**	96.7 ± 20.5	1.8 ± 1.6	0.4 ± 1.8	0.8 ± 0.6	0.0 ± 0.1	-	0.4 ± 0.1
**3**	95.9 ± 31.8	1.6 ± 0.8	-	0.4 ± 0.5	1.5 ± 2.0	-	0.6 ± 2.1
**3a**	93.5 ± 7.2	3.5 ± 0.5	-	-	2.7 ± 1.8	-	0.3 ± 0.2
**3b**	88.5 ± 43.4	4.9 ± 1.8	-	0.8 ± 1.1	5.0 ± 2.8	-	0.8 ± 2.3
**4**	94.1 ± 27.9	1.9 ± 0.9	0.1 ± 0.4	1.1 ± 1.3	1.7 ± 0.9	-	1.0 ± 1.0
**5**	97.4 ± 51.6	0.3 ± 0.8	-	0.3 ± 0.8	1.4 ± 1.6	-	0.6 ± 0.5
**6**	88.6 ± 111.8	1.1 ± 2.1	0.8 ± 2.0	-	7.1 ± 16.8	-	2.4 ± 16.7
**7**	80.7 ± 27.5	13.5 ± 12.1	-	0.1 ± 0.3	4.2 ± 2.3	-	1.4 ± 2.2
**8**	97.5 ± 48.2	1.0 ± 0.9	-	-	1.1 ± 1.1	-	0.4 ± 1.1
**9**	78.3 ± 45.8	9.7 ± 9.6	-	0.8 ± 0.7	10.9 ± 4.7	-	0.4 ± 0.1
**10**	70.4 ± 59.4	-	27.3 ± 22.6	-	1.1 ± 1.7	-	1.1 ± 1.7
**11**	96.8 ± 90.7	0.6 ± 0.7	-	0.3 ± 1.0	1.7 ± 3.3	-	0.6 ± 3.3
**12**	71.4 ± 23.3	1.7 ± 1.2	-	0.1 ± 0.2	23.6 ± 19.7	0.6 ± 1.3	2.6 ± 18.6

Table S2.2. Sinking fraction composition per category.

Sinking fraction	SIROpad		IR
1 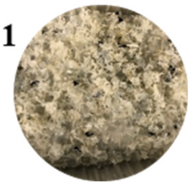	PET<br>PP<br>PE<br>Black flakes<br>Cellulose	98.61 %<br>0.62 %<br>0.07 %<br>0.68 %<br>0.01 %	PET<br>PE<br>Black flakes
2 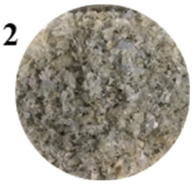	PET	100 %	PET
3 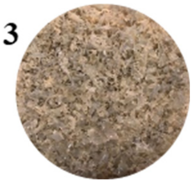	PET	100 %	PET<br>Wood fibers residue
3a 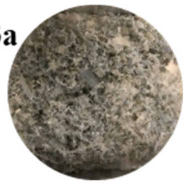	PET	100 %	PET<br>Paper<br>Wood fibers residue
3b 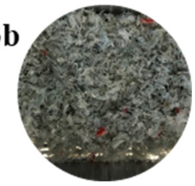	PET<br>PE<br>Black flakes<br>Cellulose	99.72 %<br>0.22 %<br>0.01%<br>0.05 %	PET<br>PE<br>Black flakes

4 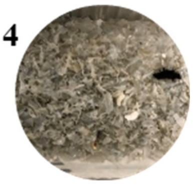	PET<br>PP<br>PE<br>Low<br>Cellulose	99.70 %<br>0.18 %<br>0.01 %<br>0.09 %<br>0.02 %	PET<br>PE
5 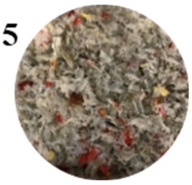	PET<br>PP<br>PE<br>Cellulose	96.83 %<br>0.02 %<br>3.14 %<br>0.01 %	PET<br>PE
6 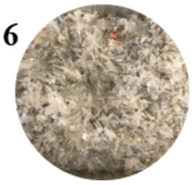	PET<br>PP<br>PE	99.82 %<br>0.02 %<br>0.16 %	PET
7 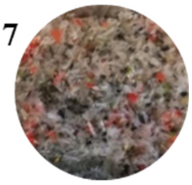	PET<br>PP<br>PE	87.42 %<br>0.63 %<br>11.95 %	PET<br>PE
8 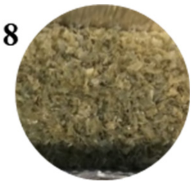	PET<br>PE	99.998 %<br>0.002 %	PET
9 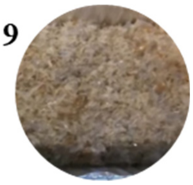	PET<br>PP<br>PE<br>PS	93.44 %<br>0.68 %<br>5.46 %<br>0.41 %	PET

10 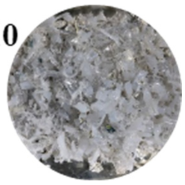	PET<br>PP<br>PE<br>PVC<br>Low	99.90 %<br>0.02 %<br>0.01 %<br>0.064 %<br>0.006 %	PET<br>PCL capa6500
11 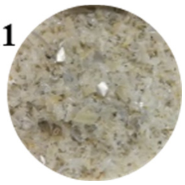	PET<br>PP<br>PE<br>Low<br>Cellulose	99.22 %<br>0.07 %<br>0.67 %<br>0.02 %<br>0.02 %	PET<br>Wood fibers resi-<br>due
12 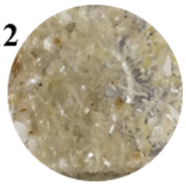	PET<br>PET (white)<br>Cellulose	93.47 %<br>6.07 %<br>0.46 %	PET

Table S2.3. Yields of laboratory-scale recycling per category.

Category	Rm [%]	c^{PET} $_{sinking\ fraction}$ [%]	$c^{PET}{}_{feedstock}$ [%]	ηPET [%]
**1**	60	98.61	74.60	91.84
**2**	92	100	97.24	89.40
**3**	89	100	96.66	91.08
**3a**	76	100	94.82	75.97
**3b**	79	99.72	89.30	89.74
**4**	81	99.70	94.36	81.85
**5**	82	96.83	90.30	90.08
**6**	84	99.82	88.78	91.74
**7**	81	87.42	69.95	92.21
**8**	93	100	97.68	84.95
**9**	67	93.44	78.50	79.07
**10**	82	99.90	99.36	86.74
**11**	83	99.22	97.13	90.07
**12**	59	93.47	72.08	75.70

Table S2.4. Intrinsic viscosity and molecular weight values of PET flakes after washing per category.

Category	IV [dl/g]	Mw (×10^3) [g/mol]
**1**	0.62 ± 0.06	32.22 ± 4.28
**2**	0.63 ± 0.06	33.26 ± 4.26
**3**	0.60 ± 0.02	30.49 ± 1.73
**3a**	0.60 ± 0.03	30.83 ± 2.04
**3b**	0.61 ± 0.04	31.36 ± 2.87
**4**	0.60 ± 0.03	30.50 ± 2.01
**5**	0.62 ± 0.03	32.07 ± 1.93
**6**	0.66 ± 0.02	35.56 ± 1.73
**7**	0.61 ± 0.01	30.98 ± 0.86
**8**	0.58 ± 0.01	28.65 ± 0.80
**9**	0.60 ± 0.02	30.70 ± 1.26
**10**	0.70 ± 0.01	38.74 ± 0.43
**11**	0.57 ± 0.02	28.06 ± 1.39
**12**	0.62 ± 0.003	31.98 ± 0.25

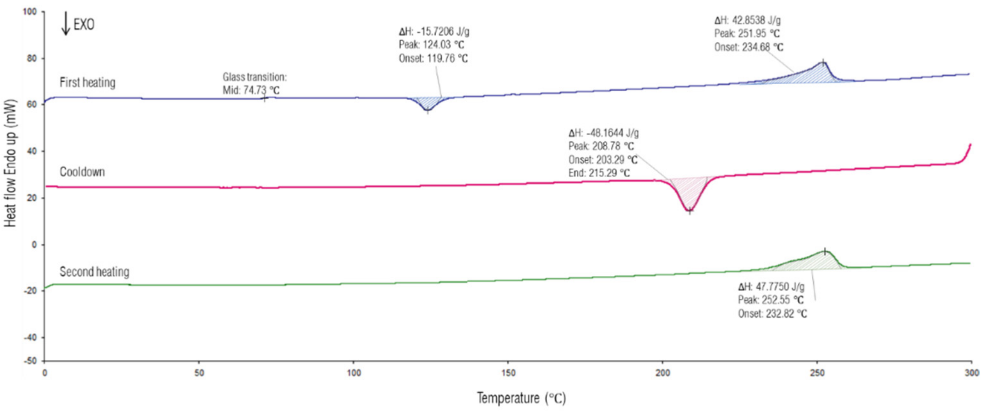

Figure S2.1 *Thermogram of DSC measurements of a sample of the 12th category*

In this study, the degree of crystallinity of each sample analyzed has been deduced from the area under the melting peak observed in the second heating run of the DSC measurement (green curve). This run is defined by controlled conditions and is indicative of the morphological properties of the sample. Trought comparing the area under the melting peak to the theoretical melt enthalpy (135,5 J/g for PET), it was possible to estimate each sample's crystallization degree. Table S2.5 indicates the derived degrees of crystallization for the different categories, ranging from 30% to almost 38%. The differences are not very high; however, these values have been used in the MCDA to compare the categories. The glass transition temperature Tg values show slight variation, ranging from 74 to 82 °C. The Tg values for the samples are between the minimum and maximum Tg values of the virgin PET, which are 69 - 115 °C.

Table S2.5. DSC data for category: melting temperature, onset of cooling temperature, degree of crystallinity values and glass transition temperature.

Category	T_m (°C)	T_m onset (°C)	X_c (%)	T_g (°C)
**1**	250 ± 2	201 ± 6	34 ± 3	76 ± 2
**2**	248 ± 4	198 ± 4	38 ± 7	79 ± 0.3
**3**	249	208	33	78
**3a**	252	204	33	79
**3b**	250	201	32	78
**4**	253	202	33	81 ± 2
**5**	251 ± 2	201 ± 2	30 ± 3	77 ± 2
**6**	253 ± 1	205 ± 10	32 ± 1	74 ± 2
**7**	251 ± 1	205 ± 5	32 ± 6	77 ± 2
**8**	249 ± 1	205 ± 0.2	34 ± 1	78 ± 1

**9**	249.3± 0.2	198 ± 1	34.5 ± 0.5	75 ± 8
**10**	252.1± 0.5	191 ± 3	34	78 ± 3
**11**	251.9 ± 1.5	201 ± 0.5	34 ± 5	80 ± 1
**12**	251 ± 2	197 ± 8	34 ± 1	74

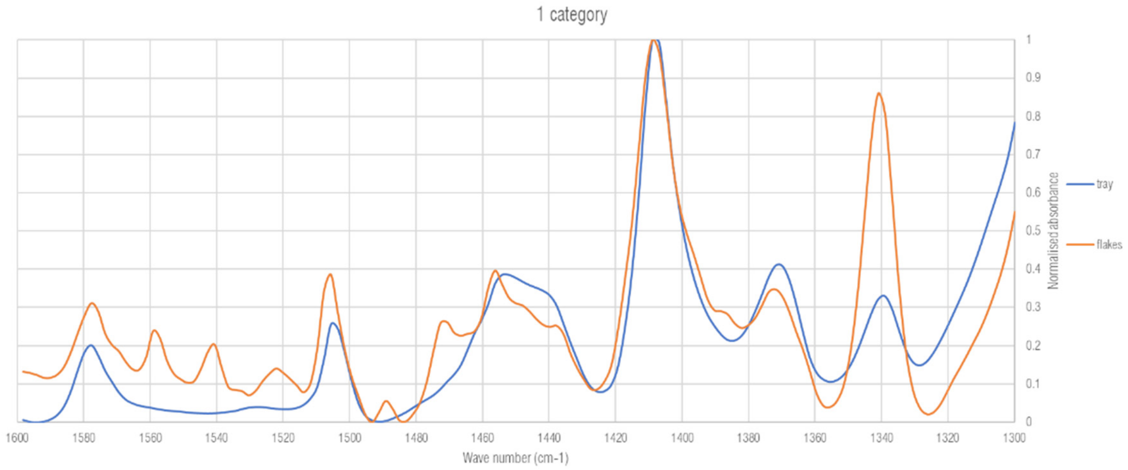

Figure S2.2 Spectra comparison between tray and flake samples wavelength 1320-1520 cm-1 of a sample of the first category

IR spectra have been analyzed to evaluate both qualitatively and quantitatively the sample's crystallization degree and assess the sample's degradation caused by the mechanical process. Hence, by examining the intensities of the peaks at precise wavelengths, it is possible to assess the degree of crystallinity of the sample. For example, the 1340 cm-1 band corresponds to the CH2 wagging vibration mode of the trans conformers of the ethylene glycol moiety. A shift in the peak position to a higher wavenumber (1342 cm-1) indicates that the ethylene glycol segment is present in a highly ordered structure, such as in thermally crystallised PET. As shown in Figure S2.2 as an example, the spectrum showed characteristic peaks of increased crystallinity at these wavelengths for flake samples. Indeed, almost all categories presented increases in peak intensity at 1470 cm-1 linked to the crystallisation, which showed up by trans conformation of the ethylene glycol segment.

All categories showed a peak closer to 726 cm-1, so the crystallinity was already detectable in the tray sample (Cole et al., 2002). Moreover, all flakes' samples show increases in peak intensity at 845 and 973 cm-1 related to the trans conformation of the ethylene glycol segment that occurs during crystallization, which can be attributable to the hot-washing step.

The fT values for the PET trays category are reported in Table S2.6, both trays and flake samples.

Table S2.6. Values of f_T detected from IR spectra for tray and flakes samples of the 14 PET tray categories.

**Category**	f_T (tray)	f_T (flakes)
**1**	0.12 ± 0.02	0.27 ± 0.01
**2**	0.11 ± 0.01	0.22 ± 0.01
**3**	0.106 ± 0.001	0.12 ± 0.01
**3a**	0.14 ± 0	0.25 ± 0.01
**3b**	0.13 ± 0.02	0.50 ± 0.04
**4**	0.12 ± 0.02	0.21 ± 0.01
**5**	0.11 ± 0.05	0.28 ± 0.02
**6**	0.11 ± 0.01	0.27 ± 0.02
**7**	0.12 ± 0.01	0.24 ± 0.01
**8**	0.10 ± 0.01	0.28 ± 0.02
**9**	0.10 ± 0.02	0.25 ± 0.01
**10**	0.23 ± 0.12	0.26 ± 0.01
**11**	0.10 ± 0.02	0.28 ± 0.02
**12**	0.14 ± 0.08	0.17 ± 0.01

Table S2.7. Results of L*a*b* and haze values referred to mono A PET tray (Bliston) of compression moulded foil per category.

Category	H	L*	a*	b*	ΔE values	YI	ΔH	ΔE
**Reference**	20.4 ± 0.6	95.83 ± 0.04	-0.13 ± 0.01	0.57 ± 0.02	95.8 ± 0.1	0.85 ± 0.02		
**1**	82 ± 21	94.7 ± 0.2	-0.4 ± 0.1	2.3 ± 0.5	94.8 ± 0.6	3.5 ± 0.8	62 ± 21	1.1 ± 0.5
**2**	89 ± 13	94.8 ± 0.4	-0.42 ± 0.04	2.5 ± 0.4	94.8 ± 0.5	3.8 ± 0.6	69 ± 14	1.0 ± 0.5
**3**	56 ± 4	94.8 ± 0.1	-0.42 ± 0.05	1.7 ± 0.1	94.8 ± 0.2	2.6 ± 0.2	35 ± 4	1.0 ± 0.2
**3a**	73 ± 6	94.9 ± 0.2	-0.5 ± 0.1	2.1 ± 0.2	94.9 ± 0.3	3.2 ± 0.2	52 ± 6	0.9 ± 0.3
**3b**	99 ± 20	94.5 ± 0.3	-0.59 ± 0.05	2.8 ± 0.5	94.6 ± 0.6	4.3 ± 0.8	78 ± 20	1.3 ± 0.6
**4**	42 ± 2	95.42 ± 0.04	-0.19 ± 0.02	1.2 ± 0.1	95.4 ± 0.1	1.85 ± 0.12	22 ± 2	0.4 ± 0.1
**5**	70 ± 13	95.0 ± 0.2	-0.4 ± 0.1	2.0 ± 0.4	95.1 ± 0.4	3.05 ± 0.53	50 ± 13	0.8 ± 0.4
**6**	43 ± 5	95.9 ± 0.1	-0.2 ± 0.1	1.3 ± 0.2	95.9 ± 0.3	1.9 ± 0.3	23 ± 5	0.03 ± 0.3
**7**	168 ± 32	94.8 ± 0.7	-1.3 ± 0.3	5.0 ± 0.9	95.0 ± 1.2	7.5 ± 1.4	148 ± 32	0.9 ± 1.2
**8**	86 ± 27	95.3 ± 0.8	-0.6 ± 0.2	2.5 ± 0.7	95.4 ± 1.1	3.8 ± 1.1	66 ± 27	0.5 ± 1.1
**9**	140 ± 61	95.2 ± 0.7	-0.9 ± 0.3	4.1 ± 1.7	95.3 ± 1.9	6.13 ± 2.62	120 ± 61	0.6 ± 1.9
**10**	25 ± 4	96.2 ± 0.1	-0.09 ± 0.05	0.8 ± 0.1	96.2 ± 0.2	1.13 ± 0.22	5 ± 4	0.4 ± 0.2
**11**	32 ± 4	96.0 ± 0.1	-0.15 ± 0.02	1.0 ± 0.1	96.0 ± 0.1	1.4 ± 0.1	12 ± 4	0.2 ± 0.1
**12**	75 ± 33	96 ± 1	-0.3 ± 0.1	2.2 ± 0.9	96.1 ± 1.4	3.2 ± 1.3	55 ± 33	0.3 ± 1.4

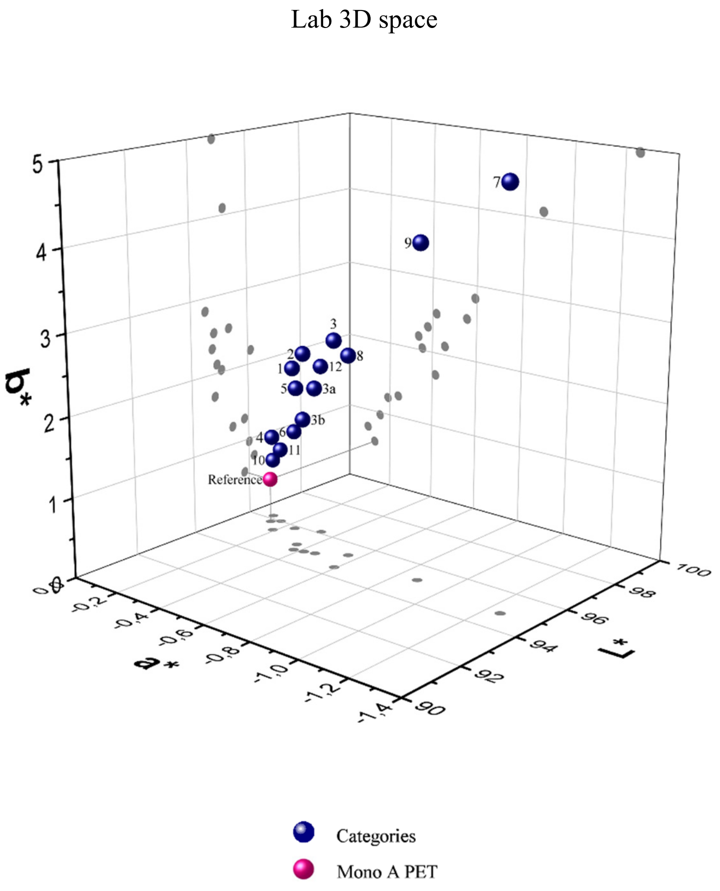

Figure S2.3. Results of L*a*b* and haze values referred to mono A PET tray (Bliston) of compression moulded foil per category.

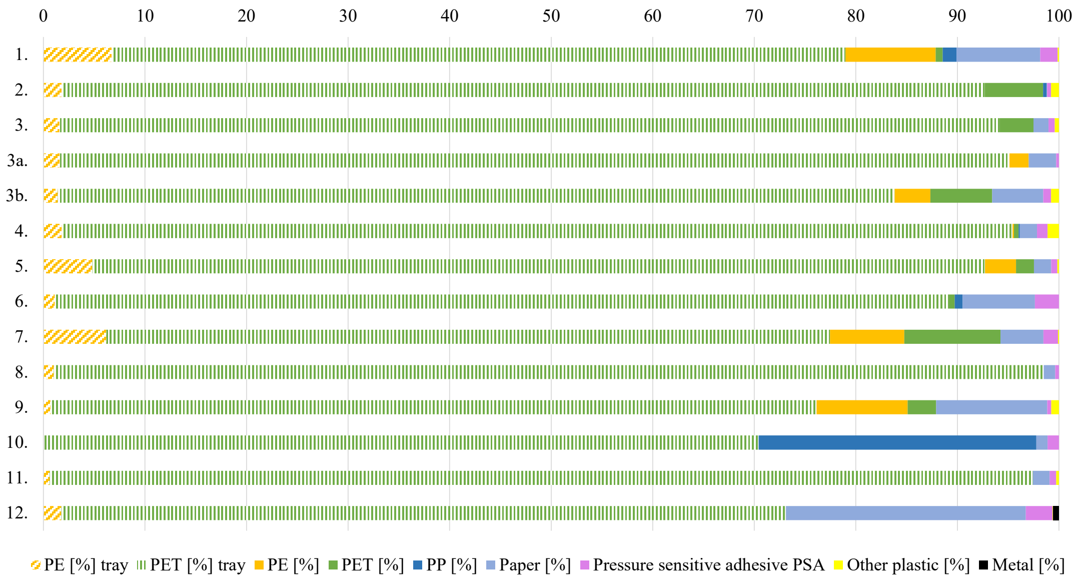

a)

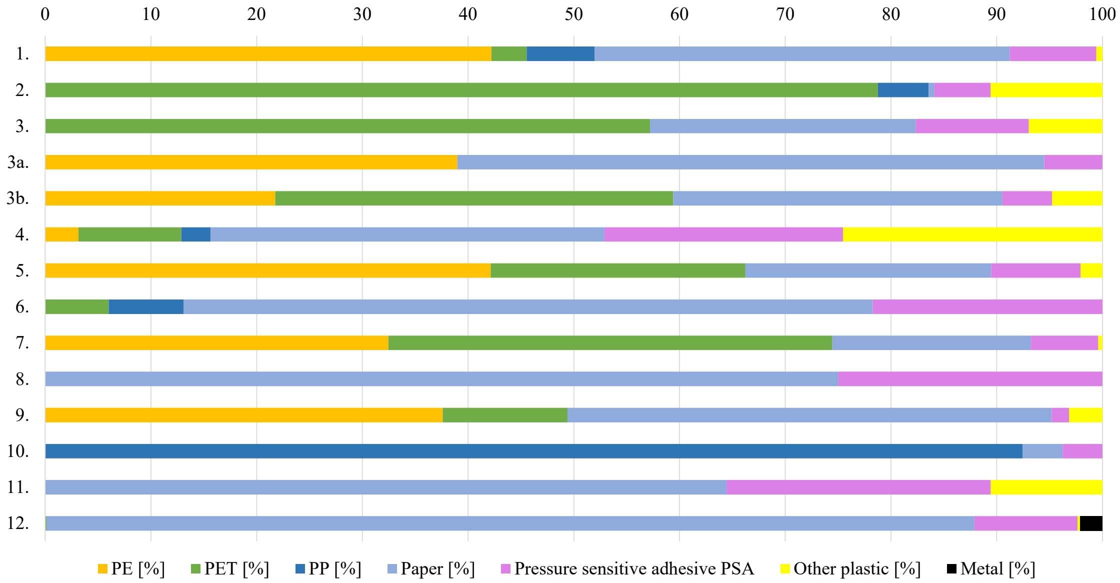

b)

Figure S2.4. a) Average material composition of total package for each category [% m/m]; b) Average material composition of components for each category [% m/m].

Table S2.8. Global weights for recycling assessment of the 14 categories.

Category	Global weights (1st scenario)	Global weights (2nd scenario)	Global weights (3rd scenario)
**1**	0.45	0.45	0.45
**2**	0.52	0.52	0.52
**3**	0.61	0.61	0.61
**3a**	0.51	0.49	0.50
**3b**	0.47	0.52	0.48
**4**	0.63	0.61	0.62
**5**	0.50	0.51	0.51
**6**	0.68	0.68	0.67
**7**	0.42	0.48	0.46
**8**	0.54	0.52	0.53
**9**	0.34	0.38	0.37
**10**	0.66	0.63	0.64
**11**	0.66	0.62	0.63
**12**	0.49	0.50	0.50

REFERENCES

Santomasi G.; Aquilino R.; Brouwer M.; De Gisi S.; Smeding I.; Todaro. F.; Notarnicola M.; Thoden van Velzen E.U. Strategies to Enhance the Circularity of Non-Bottle PET Packaging Waste Based on a Detailed Material Characterisation. **2024**, doi:10.1016/j.wasman.2024.06.016.

Thoden van Velzen, E.U.; Brouwer, M.T.; Molenveld, K. Technical Quality of RPET : Technical Quality of RPET That Can Be Obtained from Dutch PET Bottles That Have Been Collected, Sorted and Mechanically Recycled in Different Manners; 2016; ISBN 9789462577237.

Badía, J.D.; Vilaplana, F.; Karlsson, S.; Ribes-Greus, A. Thermal Analysis as a Quality Tool for Assessing the Influence of Thermo-Mechanical Degradation on Recycled Poly(Ethylene Terephthalate). Polym. Test. 2009, 28, 169–175, doi:10.1016/j.polymertesting.2008.11.010.

Candal, M.V.; Safari, M.; Fernández, M.; Otaegi, I.; Múgica, A.; Zubitur, M.; Gerrica-Echevarria, G.; Sebastián, V.; Irusta, S.; Loaeza, D.; et al. Structure and Properties of Reactively Extruded Opaque Post-Consumer Recycled PET. Polymers (Basel). 2021, 13, doi:10.3390/polym13203531.

Cole, K.C.; Ajjii, A.; Pellerin, É. New Insights into the Development of Ordered Structure in Poly(Ethylene Terephthalate). 1. Results from External Reflection Infrared Spectroscopy. Macromolecules 2002, 35, 770–784, doi:10.1021/ma011492i.

Badia, J.D.; Strömberg, E.; Karlsson, S.; Ribes-Greus, A. The Role of Crystalline, Mobile Amorphous and Rigid Amorphous Fractions in the Performance of Recycled Poly (Ethylene Terephthalate) (PET). Polym. Degrad. Stab. 2012, 97, 98–107, doi:10.1016/j.polymdegradstab.2011.10.008.

Dubelley, F.; Planes, E.; Bas, C.; Pons, E.; Yrieix, B.; Flandin, L. The Hygrothermal Degradation of PET in Laminated Multilayer. Eur. Polym. J. 2017, 87, 1–13, doi:10.1016/j.eurpolymj.2016.12.004.

Kong, Y.; Hay, J.N. The Measurement of the Crystallinity of Polymers by DSC. Polymer (Guildf). 2002, 43, 3873–3878, doi:10.1016/S0032-3861(02)00235-5.

Olagoke Olabisi; Kolapo Adewale Handbook of Thermoplastics; CRC Press, Ed.; 2nd ed.; Plastics Engineering, 2016; Vol. 41; ISBN 1466577231, 9781466577237.

Bashir, Z.; Al-Aloush, I.; Al-Raqibah, I.; Ibrahim, M. Evaluation of Three Methods for the Measurement of Crystallinity of PET Resins, Preforms, and Bottles. Polym. Eng. Sci. 2000, 40, 2442–2455, doi:10.1002/pen.11376.

Demirel, B.; Yaraş, A.; Elçiçek, H. Crystallization Behavior of PET Materials; 2011; Vol. 13.
